# Aromatase Inhibitors Plus Weight Loss Improves the Hormonal Profile of Obese Hypogonadal Men Without Causing Major Side Effects

**DOI:** 10.3389/fendo.2020.00277

**Published:** 2020-05-15

**Authors:** Georgia Colleluori, Rui Chen, Christie G. Turin, Francesca Vigevano, Clifford Qualls, Biju Johnson, Sanjay Mediwala, Dennis T. Villareal, Reina Armamento-Villareal

**Affiliations:** ^1^Division of Endocrinology, Diabetes and Metabolism, Baylor College of Medicine, Houston, TX, United States; ^2^Center for Translational Research on Inflammatory Diseases, Michael E. DeBakey VA Medical Center, Houston, TX, United States; ^3^Division of Mathematics and Statistics, University of New Mexico School of Medicine, Albuquerque, NM, United States; ^4^Research Pharmacy, Michael E. DeBakey VA Medical Center, Houston, TX, United States

**Keywords:** hypogonadism, obesity, aromatase inhibitors, weight loss, sex hormones, bone density, bone microarchitecture, body composition

## Abstract

**Objective:** In obese men, the increased expression of the aromatase enzyme in adipose tissue leads to high conversion of androgens to estrogens contributing to hypogonadotropic hypogonadism (HHG). Our objective is to evaluate efficacy and safety of weight loss (WL) plus aromatase inhibitor (AI) therapy in severely obese men with HHG. We hypothesize that AI+WL will be more effective as compared to WL alone in improving the hormonal profile, thus muscle strength and symptoms of HHG (primary outcomes), with no significant adverse effects on lean mass, metabolic profile, and bone mineral density (secondary outcomes).

**Design:** Randomized double-blind placebo-controlled pilot trial.

**Methods:** Twenty-three obese men (BMI≥35 kg/m^2^), 35–65 years old, were randomized to weight loss (diet and exercise) plus either anastrozole (AI+WL, *n* = 12) at 1 mg daily or placebo (PBO+WL, *n* = 11) for 6 months. Inclusion criteria: total testosterone <300 ng/mL (average of 2 measurements), estradiol≥10.9 pg/ml, LH <9 IU/l. Symptoms of hypogonadism by questionnaires; muscle strength by Biodex dynamometer; body composition and bone mineral density by dual-energy X-ray absorptiometry; bone microarchitecture and finite element analysis by high resolution peripheral quantitative-computed tomography.

**Results:** After 6 months of therapy, AI+WL group had higher testosterone (*p* = 0.003) and lower estradiol (*p* = 0.001) compared to PBO+WL. Changes in symptoms and muscle strength did not differ between groups. AI+WL resulted in higher fat mass loss than PBO+WL (*p* = 0.04) without differences in changes in lean mass. Total and LDL cholesterol reduced more in the PBO+WL group compared to AI+WL (*p* = 0.03 for both), who experienced a minimal increase with unlikely meaningful clinical impact. Tibial trabecular bone area decreased more in PBO+WL than AI+WL group for which it remained stable (*p* = 0.03).

**Conclusions:**Although AI+WL is effective in reversing the hormonal profile of HHG in severely obese men without causing major side effects, it does not lead to greater improvements in muscle strength and symptoms of hypogonadism compared to WL alone.

**Clinical Trial Registration**: www.ClinicalTrials.gov, identifier: NCT02959853.

## Introduction

The prevalence of hypogonadism among obese men ranges from 29.3% (1) to 78.8% (2), with levels of androgens decreasing proportionately with the degree of obesity (3, 4). The increased expression of the aromatase enzyme in the adipose tissue of obese men leads to a greater conversion of androgens to estrogen (5). The consequent hyperestrogenemia exerts a negative feedback on the hypothalamic-pituitary-gonadal axis, with inhibition in the production of luteinizing hormone (LH) and follicular stimulating hormone (FSH), resulting in reduced testosterone output from the testes leading to what has been described as hypogonadotropic hypogonadism (HHG) (6, 7). Due to the high aromatase expression in the adipose tissue, it is possible that the administration of testosterone in obese men with HHG will only perpetuate the cycle of obesity and hypogonadism rather than correct the underlying problem. Our preliminary unpublished data (presented as an abstract at the annual Endocrine Society meeting in 2016) showed no improvement in lean mass or hypogonadal symptoms in severely obese men (with body mass index [BMI]≥35 kg/m^2^) compared to subjects with lower BMI after 12 months of testosterone cypionate injections. It is thus possible that men with obesity induced HHG benefit from treatment strategies that target the underlying mechanism of the disease.

According to a metanalysis, weight loss (WL) by lifestyle modifications or surgical intervention improve the hormonal profile of obese men with HHG (8). However, these benefits are often lost due to weight regain (9), which is very frequent among patients undergoing massive WL (10). In addition, the improvement in total testosterone (TT) level is quite limited and not sufficient to reach the target level (usually mid-range of normal ~500 ng/dL) (11). One possible alternative approach is the use of aromatase inhibitors (AI) to inhibit the conversion of androgens into estrogens, thereby interrupting the cycle of the estrogen-induced inhibition of the hypothalamic-pituitary-gonadal axis. Testosterone increase and estradiol (E2) reduction have been reported in hypogonadal men treated with AI (6, 12–16). However, only a limited number of studies have investigated the effect of AI therapy on clinical outcomes; thus, the safety and efficacy on the use of AIs in men with obesity-induced HHG remains largely undetermined (6, 12–18). Because WL remains the standard of care in obesity, we designed this proof-of-concept study to determine the efficacy and safety of the combination of WL plus AI in severely obese men with HHG. The objectives of this study are to evaluate the effects of AI plus WL (AI+WL) compared to placebo plus WL (PBO+WL) on the hormonal profile and on muscle strength and symptoms of hypogonadism (primary outcomes). Our secondary (which include safety) outcomes are to evaluate changes in body composition, bone health, and metabolic markers. We hypothesize that AI+WL will be more effective as compared to WL alone in improving the hormonal profile, thus muscle strength and symptoms of HHG, with no significant adverse effects on lean mass, metabolic profile, and bone mineral density (BMD).

## Materials and Methods

### Study Design

This is a randomized, double-blind, placebo-controlled pilot study comparing WL plus AI vs. WL plus PBO. The study was conducted at the Michael E. DeBakey VA Medical Center (MEDVAMC) in accordance with the guidelines in the Declaration of Helsinki for the ethical treatment of human subjects. The protocol was approved by the Baylor College of Medicine Institutional Review Board (ClinicalTrials.gov Identifier: NCT02959853). The original (Supplement 1) protocol is available online. Pre-specified and *post-hoc* outcome measures are summarized in Supplement 1. Considering the pilot nature of this clinical trial, sample power calculation was not performed. Data collected from this study will be used to obtain sample power calculation for the design of a bigger trial.

### Study Participants

Public advertisements were used in the MEDVAMC to recruit suitable patients. One-hundred ninety-five potential study candidates underwent a screening evaluation including medical history, physical examination, and laboratory tests. Male Veterans between 35 and 65 years of age, severely obese (BMI≥35 kg/m^2^), with HHG [defined by total TT <300 ng/dl, LH <9 IU/l, and E2 ≥10.9 pg/ml as reported by Loves et al. (14)] were eligible for enrollment. Potential participants were excluded if they had pituitary, heart, liver, renal disease, or any medical condition that could interfere with bone metabolism. Those participants with history of bariatric surgery, severe lower urinary tract symptoms (International Prostate Symptom Score > 19), excessive alcohol or substance abuse or use of any medication that could affect gonadal hormones, steroid hormone-binding globulin levels, and bone metabolism were also excluded. Prostate-specific antigen (PSA) >4 or >3 ng/ml for subject with family history of prostate cancer, hematocrit ≥50%, and elevated liver enzymes more than twice the upper limit of normal were also considered exclusion criteria. All patients provided written informed consent.

### Aromatase Inhibitor Therapy

Participants were randomized by the MEDVAMC pharmacy from a list generated by a research biostatistician to one of two treatment groups for a total of 26 weeks: AI (anastrozole 1 mg daily) + WL or PBO + WL. The MEDVAMC research pharmacist was responsible for dispensing the study drugs on a monthly basis and for keeping a record of all dispensed medications. In order to assure blinding, randomization was performed by the study statistician who forwarded the randomization list to the research pharmacist who in-turn dispensed the study drug directly to the subjects. A designated unblinded investigator who was not involved in the outcome measurements was responsible for adjusting the dose of the study drug. The rest of the research team including the principal investigator, research coordinator, dietitian, and exercise physiologist were blinded as to the randomization of the participants until the end of the study.

### Weight Loss Intervention

All individuals participated in a WL program consisting of a diet plan and unsupervised exercise monitored weekly by meetings with a dietitian. Diet plan was individualized for each patient to provide an energy deficit of 500–750 kcal/day from daily caloric intake containing ~1 g of high-quality protein/kg/day. The goal was to achieve a reduction of ~10% of their baseline weight within 6 months of intervention. All participants were also asked to perform at least 150 min/week of unsupervised moderately intense exercise, as suggested by the American College of Sports Medicine (ACSM). Each participant received a pedometer to track their exercise level (#HJ-321, OMRON HEALTHCARE, INC. Lake Forest, IL 60045 USA). During the weekly meetings with the dietitian, food diaries, behavioral goals, and pedometer results were revised for adjustments of their caloric intake and physical activity.

### Hormonal Profile

Initial measurement of TT and E2 was performed by immunoassay at the MEDVAMC laboratory. The TT level used for screening was the average of 2 samples collected between 8:00 and 10:00 a.m., 2–3 days apart [1]. TT was measured using an automated immunoassay, detection range 10–3200 ng/dl (Vitros®, Ortho Clinical Diagnostics, Rochester, NY). The coefficient of variability for this assay is ≤ 20% for testosterone levels of <50 ng/dl, and ≤ 10% for testosterone levels between 200 and 1000 ng/dl. At the end of the study, TT and estradiol for each timepoint were measured by liquid chromatography/mass spectrometry (Mayo Clinic Laboratories, Mayo Clinic, Rochester, MN). TT intra-assay CVs are 7.4, 6.1, 9.0, 2.3, and 0.9% at 0.65, 4.3, 48, 118, and 832 ng/dl, respectively. Inter-assay CVs are 8.9, 6.9, 4.0, 3.6 and 3.5% at 0.69, 4.3, 45, 117, and 841 ng/dl, respectively. The detection range is 0.5–2,000 ng/dl. Estradiol assay sensitivity is 0.23–405 pg/ml, intra-assay CV is 1.4–11.8% and inter-assay CV is 4.8–10.8% (19). Measurement of FSH and LH was performed at the MEDVAMC clinical laboratory. FSH was measured by immunoenzymatic assay with the UNICEL Dxl 600 Immuoassay System (Beckman Coulter, Inc., 250 S. Kraemer Blvd., Brea, CA 92821 USA); detection limit is 0.2–200 IU/l; CV <10%. LH was measured by immunoenzymatic assay with the UNICEL Dxl 600 Immuoassay System (Beckman Coulter, Inc., 250 S. Kraemer Blvd., Brea, CA 92821 USA); detection limit is 0.2–250 IU/l; CV <10%. For follow-up laboratory testing required at 3 and 6 months of intervention, blood samples were collected in the morning (between 8 a.m. and 11 a.m.) before anastrozole ingestion.

### Muscle Strength Testing

Muscle strength was assessed using Biodex System 4 Isokinetic Dynamometer (Shirley, NY). Peak torque for isokinetic knee extension and flexion was measured at baseline, 3 and 6 months on the right leg. During the testing, participants sat with their hips flexed at 120°, secured with thigh and pelvic straps. Testing was performed at an angular velocity of 60° per second. The best result of 3 maximal voluntary efforts for each knee flexion and extension was used as the measure of absolute strength and reported as peak torque at 60° in Newton-meter (N^*^m) units. Using this method, the test–retest reliability based on follow-up testing 1 week after the initial tests showed an intra-class correlation coefficient of 0.99 (20, 21).

### Evaluation of Symptoms of Hypogonadism

Participants filled 3 validated questionnaires at baseline, 3 and 6 months to assess symptoms of hypogonadism and quality of life. The quantitative Androgen Deficiency in the Aging Male (qADAM) (22) questionnaire is a variant of the ADAM questionnaire (widely-used as screening tool for androgen deficiency) that replaced the “yes” or “no” answer by a scale of 1–5, in which 1 represents maximal symptoms and 5 represents absence of symptoms. The final summation of the responses yields a total score between 10 (most symptomatic) and 50 (least symptomatic). The second questionnaire used was the International Index of Erectile Function (IIEF)-5 (23), which is a reduced version of one of the most frequently used tools to assess male sexual function (24). It consists of 5 questions that evaluate erectile function, orgasmic function, sexual desire, intercourse satisfaction, and overall satisfaction. Total score ranges from 5 to 25, 1–7 being severe erectile dysfunction (ED) and 22-25 no ED (23). The third questionnaire used was the Impact of Weight on Quality of Life Questionnaire-Lite (IWQOL) to evaluate the effect of weight on health-related quality of life (25). The IWQOL is composed by 5 scales: physical function, self-esteem, sexual life, public distress and work. Scores per question range from 5 (worst symptomatology) to 1 (asymptomatic). For safety monitoring, we also assessed changes in urinary symptoms using the validated International Prostate Scoring System (IPSS). In addition, the Stanford 7-day Physical Activity Recall (PAR) was used to monitor changes in physical activity and ensure an active lifestyle. Information regarding weekly smoking and alcohol consumption were also collected. A copy of all questionnaires is reported in Supplement 2.

### Body Mass Index and Body Composition

Body weight and height were measured by standard weighing scale and stadiometer, respectively. BMI (kg/m^2^) was calculated by dividing the weight (in kilograms) by height (in meters) squared. The body composition assessment was performed by dual-energy X-ray absorptiometry (DXA) (Hologic-Discovery; Enhanced Whole Body 11.2 software version; Hologic Inc, Bedford, MA; USA) at baseline, 3 and 6 months. Images were analyzed following manufacturer's instructions. The coefficient of variation for lean mass and fat mass measurements in our laboratory is 1.5%.

### Metabolic Profile and Biochemical Measurements

Hemoglobin A1c, lipid profile (triglycerides, LDL, and HDL cholesterol) were measured at the MEDVAMC Laboratory. In brief, hemoglobin A1c was measured by high performance liquid chromatography (HPLC) with the Tosoh Automated Glycohemoglobin Analyzer HLC-723G8. (Tosoh Bioscience, Inc. South San Francisco, CA 94080); CV <2%. Total cholesterol and triglycerides were measured by fluorometric assay and LDL and HDL were measured by colorimetric assay by UNICEL DxC (Beckman Coulter, Inc., 250 S. Kraemer Blvd., Brea, CA 92821 USA). Detection limits for these measurements are: 7–750 mg/dL (0.13–19.43 mmol/L) for total cholesterol, 11–500 mg/dl for LDL, 5–135 mg/dL (0.13–3.5 nmol/L) for HDL, 10–1,000 mg/dL (0.1–11.3 mmol/L) for triglycerides; CVs <10% for all measurements. Enzyme-linked immunosorbent assays were used to assess circulating markers of bone turnover such as osteocalcin (OC) (Metra OC; Quidel Corporation, San Diego, CA) a marker of bone formation and C-terminal telopeptide (CTX) (Immunodiagnostic System Inc., Gaithersburg, MD), a marker of bone resorption. The CVs for these assays in our laboratory are 4.4% for OC and 2.1% for CTX (26).

### Bone Mineral Density, Geometry, and Microarchitecture

The BMD of the lumbar spine and proximal femur (which included the total hip and the femoral neck) was measured by DXA at baseline and 6 months. The CVs at our center are ~1.1% for the lumbar spine and ~1.2% for the proximal femur (27).

Volumetric BMD, bone geometry, microarchitecture, and mechanical properties were measured at baseline and 6 months by High Resolution Peripheral Quantitative Computed Tomography (HRpQCT) (Xtreme CTII, Scanco Medical AG, Brüttisellen, Switzerland) at the non-dominant distal radius and tibia (or in the contralateral arm/leg in case patient had prior surgeries or experienced previous fractures on the non-dominant extremity). A stack of 168 parallel CT slices, representing a 10.2 mm length, was acquired for each site. Isotropic voxel size was 61 μm and all scans were performed according to the standard protocols: nominal high voltage of 68 kVp, X-ray tube current of 1470 μA, for total scan time of 2.0 min. Limbs of interest were immobilized in a carbon cast during the examination and a scout view was performed prior to scanning in order to identify and place a reference line on the endplate of radius and tibia. The first slice was acquired 9.0 and 22.0 mm proximal to the reference line for the radius and tibia, respectively, as per standard analysis. If motion artifacts were observed, scans were repeated up to 3 times to improve the image quality, as per manufacturer recommendation and with the agreement of the participant. To extract the mineralized bone phase, we used a low-pass Gaussian filter (sigma 0.8, support 1.0). A fixed threshold was applied to extract the trabecular bone (320 mg HA/cm^3^) and cortical bone (450 mg HA/cm^3^). We assessed microarchitecture directly in the trabecular and cortical regions using voxel-based measurements. Parameters of interest were Total Volumetric BMD –Tot.vBMD- (mg HA/cm^3^), Trabecular Volumetric BMD –Tb.vBMD- (mg HA/cm^3^), Trabecular Thickness -Tb.Th- (mm), Trabecular Number -Tb.N- (mm^−1^), and Trabecular Separation -Tb.Sp- (mm), Cortical BMD–Ct.vBMD- (mg HA/cm^3^), Cortical Thickness –Ct.Th- (mm), Cortical Porosity –Ct.Po- (unit free).

Micro-Finite Element Analysis –μFEA- of radius and tibia, which are represented by failure load and stiffness, were performed creating μFE models that represent the actual bone microarchitecture in detail, thus converting each voxel of bone tissue into an equally sized brick element. Isotropic and elastic mechanical properties were chosen. Cortical and trabecular bone elements were assigned a Young's modulus of 20 and 17 GPa, respectively, and a Poisson's ratio (28). The μFEA consists of a compression test simulation in which a load in the longitudinal direction is applied at one end, while the other end was fully constrained, to simulate a fall from standing height on an outstretched hand. The failure load was calculated using the criterion developed by Pistoia et al. Such model of bone failure load was demonstrated to well-predict experimental failure load measured by loading cadaver forearms, reproducing a fall on an outstretched hand. Stiffness is the extent to which an object resists deformation in response to an applied force. The same parameters were used for the tibia analysis, as this was shown earlier to be associated with fragility fractures. The CVs for the different parameters measured by HR-pQCT are as follows: 0.2–2.5% for geometry, 0.6–1.7% for BMD, 0.7–2.4% for trabecular bone compartment parameters, and 1.1%−1.3% for cortical thickness, while cortical porosity was high at 11.0–13.3% (29).

### Statistical Analysis

Analyses of covariance (ANCOVA) adjusting for covariates was performed to compare the effect of AI+WL vs. PBO+WL on multiple variables. Specifically, we compared the difference in changes in the investigated variables between our two intervention groups. Where changes were not normally distributed (tested by Shapiro-Wilk), the non-parametric Wilcoxon test between study arms verified the results reported. All outcomes were adjusted for baseline value. Data were presented as means ± SD in tables, text and figures. Data were managed using Excel 2013 (Microsoft, Redmond, WA) and analyzed using SAS version 9.3 (SAS Institute, Inc., Cary, NC, USA). A *p* < 0.05 was considered statistically significant. Intention-to-treat analysis was performed with the last value carried forward for those who did not finished the 6-month study period but that had at least one follow-up data.

## Results

**Figure 1:**
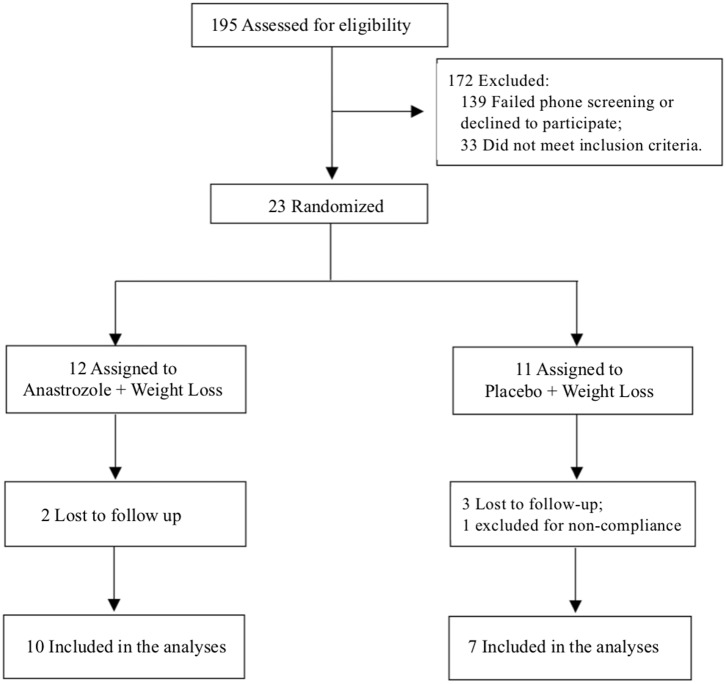
Consort diagram.

One-hundred ninety-five subjects were screened, and 23 participants were included in the study. Twelve participants were randomized to the anastrozole group and 11 to the placebo group. Seventeen completed the 6-month study, 10 in the AI+WL arm and 7 in the PBO+WL arm. Five individuals dropped out from the study, while one patient was asked to leave the study because of non-compliance to the dietary prescription (Figure 1). None of the participants dropped out because of side effects related to the intervention. Among the individuals who did not finish the 6-month study, 2 completed the 3-month assessments. The process of recruitment and randomization is detailed in a Consort diagram (Figure 1).

### Baseline Characteristics

Our study population consisted of 13 African-American, 9 Caucasian and 1 Hispanic men. Mean age, weight, and BMI of study participants were 51.7 ± 6.2 (38–62) years old, 129.9 ± 22.8 (93–195) kg, and 40.6 ± 6.0 (35.0–60.0) kg/m^2^, respectively. Average serum TT was 237 ± 47.8 ng/dl by immunoassay and 280 ± 93.5 ng/dl by LC/MS, E2 was 29 ± 9.7 pg/ml, LH was 4.9 ± 2.3 IU/l and FSH was 7.1 ± 5.1 IU/l. Baseline characteristics and hormonal profile of the participants did not differ between the AI+WL and PBO+WL groups as shown in Table 1.

**Table 1:** Baseline characteristics of the participants.

**Characteristic**	**PBO+WL**	**AI+WL**	***p***
	***n* = 11**	***n* = 12**	
Age (year)	51 ± 6	52 ± 6	0.59
Height (cm)	179 ± 8	179 ± 6	0.97
Weight (kg)	132.6 ± 25.1	127.4 ± 21.3	0.60
Body mass index (kg/m2)	41.5 ± 6.9	39.6 ± 5.1	0.46
Testosterone (ng/dl)	273.1 ± 76.4	286.5 ± 111.5	0.75
Estradiol (pg/ml)	29.4 ± 11.8	28.6 ± 7.6	0.87
FSH (IU/l)	7.5 ± 6.5	6.7 ± 3.8	0.73
LH (IU/l)	5.2 ± 2.2	4.6 ± 2.4	0.51
Hemoglobin A1c (%)	7.3 ± 1.4	6.6 ± 1.6	0.27
LDL (mg/dl)	112.3 ± 42.5	111.7 ± 43.9	0.98
Total body fat (kg)	54.0 ± 16.9	51.0 ± 10.7	0.61
Trunk fat (kg)	30.3 ± 10.1	28.0 ± 7.1	0.53
Total body lean mass (kg)	79.0 ± 8.8	77.2 ± 11.8	0.68
Appendicular lean mass (kg)	36.1 ± 5.4	34.9 ± 5.7	0.61
Total hip bone mineral density (gm/cm^2^)	1.085 ± 0.135	1.101 ± 0.153	0.81
Femoral neck bone mineral density (gm/cm^2^)	0.945 ± 0.122	0.929 ± 0.153	0.79
Lumbar spine bone mineral density (gm/cm^2^)	1.077 ± 0.091	1.135 ± 0.170	0.35

*Results are presented as mean ± standard deviation*.

### Hormonal Profile

**Figure 2:**
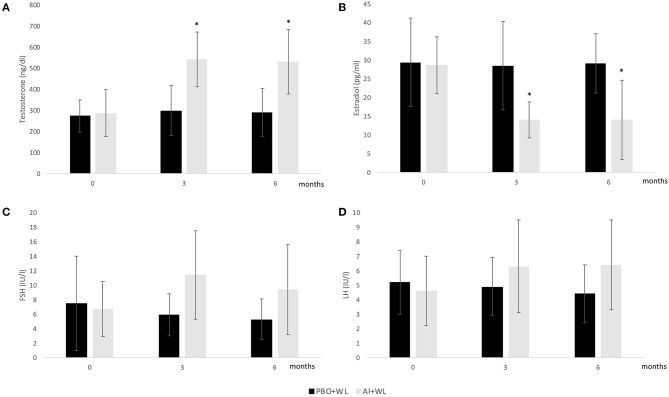
Mean serum Total Testosterone **(A)**, Estradiol **(B)**, FSH **(C)**, and LH **(D)** levels at baseline, 3 and 6 months. I bars indicates standard deviation; *p* < 0.05 for comparison with the PBO+WL group is indicated by a star; adjusted *p* values for baseline value.

The mean TT, E2, LH, and FSH after 3 and 6 months of treatment are shown in Figure 2, while changes are summarized in Table 2. As shown in this table, changes in TT were significantly different between the two study groups with the AI+WL group experiencing a greater increase compared to PBO+WL group. This resulted in higher TT levels in AI+WL vs. PBO+WL whose TT remained stable at 3 months (541.5 ± 130.1 vs. 299.3 ± 119.5 ng/dl, *p* = 0.002) and 6 months of intervention (530.0 ± 165.9 vs. 289.9 ± 132.5 ng/dl, *p* = 0.01) as shown in Figure 2. As expected, the AI+WL group experienced a significantly greater reduction in E2 levels at both timepoints (Table 2). E2 levels were lower in the AI+WL compared to PBO+WL at 3 (14.0 ± 4.8 vs. 28.5 ± 11.8 pg/mL, *p* = 0.004) and 6 months (14.0 ± 10.6 vs. 29.1 ± 7.9 pg/mL, *p* = 0.01), respectively (Figure 2). There were no significant between-group differences in the changes in LH at both time points (Table 2). While FSH levels tend to increase more in the AI+WL group at 3 months (*p* = 0.05), we did not detect significant differences between groups at the end of the study (Table 2). Absolute levels of LH and FSH at both 3 and 6 months are also shown in Figure 2. These data showed that AI+WL intervention is more effective than WL alone in reducing estradiol and increasing testosterone of patients with obesity-induced HHG.

**Table 2:** Changes from baseline in hormones at 3 and 6 months.

**Hormone**	**Change**	**PBO+WL**	**AI+WL**	***p***	**Adjusted *p***
Testosterone (ng/dl)	Δ 3 months	45.5 ± 113.2	264.4 ± 152.8	**0.01**	** <0.05**
	Δ 6 months	1.1 ± 88.5	223.9 ± 139.2	** <0.01**	** <0.05**
Estradiol (pg/ml)	Δ 3 months	−5.7 ± 16.7	−15.9 ± 7.5	0.13	**0.01**
	Δ 6 months	0.6 ± 5.9	−19.2 ± 9.9	** <0.01**	** <0.01**
FSH (IU/l)	Δ 3 months	1.1 ± 2.6	4.3 ± 3.7	**0.05**	0.11
	Δ 6 months	0.2 ± 0.8	2.3 ± 5.5	0.35	0.33
LH (IU/l)	Δ 3 months	0.4 ± 1.9	1.8 ± 2.8	0.22	0.23
	Δ 6 months	−0.3 ± 1.3	1.9 ± 3.1	0.10	0.11

*Results are presented as mean ± standard deviation; p values adjusted for baseline values. Bold p values indicate a significant between group difference*.

### Muscle Strength and Symptoms of Hypogonadism

Peak torque for isokinetic knee extension and flexion equally changed (increased) in the two groups at 6 months with no significant between-group differences (Table 3). Changes in symptoms related to androgen deficiency (qADAM), erectile dysfunction (IIEF-5), and impact of obesity on quality of life (IWQOL-lite) were not significantly different in both groups at 3 and 6 months (Table 3). Changes in lower urinary tract symptoms based on IPSS questionnaire were also not significantly different between groups at 3 and 6 months (Table 3). These data show that AI+WL is not more effective than WL alone in improving muscle strength and symptoms of hypogonadism.

**Table 3:** Change in symptoms and muscle strength at 3 and 6 months.

**Questionnaire Change at 3 and 6 months**	**Change**	**PBO+WL**	**AI+WL**	***p***	**Adjusted *p***
qADAM	Δ 3 months	2.4 ± 3.4	5.8 ± 6.1	0.16	0.09
	Δ 6 months	4.1 ± 5.7	4.4 ± 7.2	0.93	0.55
IIEF-5	Δ 3 months	4.3 ± 3.8	2.7 ± 4.6	0.46	0.69
	Δ 6 months	0.9 ± 4.1	1.7 ± 5.8	0.76	0.56
IWQOL	Δ 3 months	−12.2 ± 23.9	−13.0 ± 15.2	0.93	0.41
	Δ 6 months	−16.6 ± 27.1	−18.6 ± 8.8	0.84	0.48
IPSS	Δ 3 months	−0.9 ± 5.5	−4.5 ± 6.9	0.23	0.30
	Δ 6 months	−1.3 ± 3.9	−4.7 ± 5.2	0.18	0.30
**Change and % change in muscle strength at 6 months**					
60° Knee extension peak torque	Δ 6 months	13.5 ± 12.5	4.9 ± 16.9	0.28	0.30
	% 6 months	12.8 ± 13.8	3.7 ± 14.7	0.22	
60° Knee flexion peak torque	Δ 6 months	7.9 ± 8.3	4.4 ± 13.3	0.55	0.51
	% 6 months	12.6 ± 13.3	8.9 ± 21.6	0.70	

*Results are presented as mean ± standard deviation; p values were adjusted for baseline values*.

### Weight Loss, Body Composition, and Metabolic Markers

**Figure 3:**
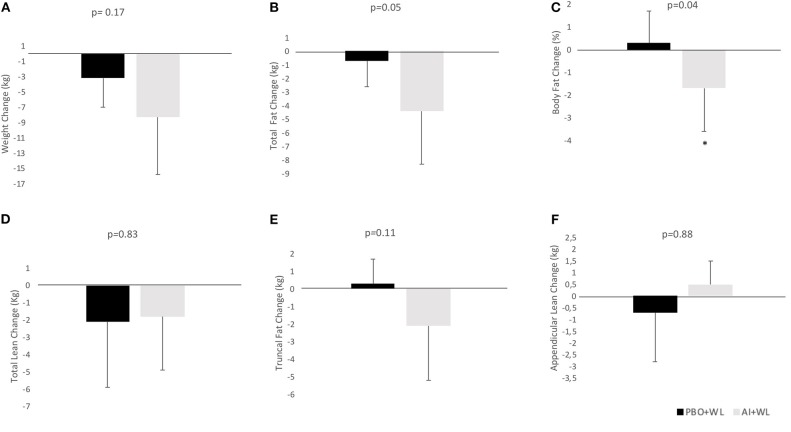
Absolute change (adjusted for baseline value) of body composition parameters at 6 months. **(A)** Weight Change; **(B)** Total Fat Change; **(C)** Body Fat Change; **(D)** Total Lean Change; **(E)** Truncal Fat Change; **(F)** Appendicular Lean Change. I bars indicated standard deviations; *p* < 0.05 is indicated by a star.

The average WL for the whole population was −6.2 ± 6.6 kg. Participants in the AI+WL group experienced greater weight reduction (−8.3 ± 7.5 kg) compared to PBO+WL group (−3.2 ± 3.8 kg) at 6 months of intervention; however, the difference did not reach statistical significance (adjusted *p* = 0.17) (Figure 3A). There was a higher reduction in total body fat mass in the AI+WL group compared to the PBO+WL group (−4.4 ± 3.9 vs. −0.7 ± 1.9 kg, *p* = 0.05) (Figures 3B,C). Mean lean mass loss for the whole population was −2.5 ± 4.3%, without significant between group differences in changes in this variable at the end of the study (Figure 3D). These data showed that AI+WL intervention could promote a higher reduction in total body fat without causing significant lean mass loss. Changes in total truncal fat and appendicular lean mass did not differ between the two groups (Figures 3E,F). Changes in hemoglobin A1c, circulating triglycerides and HDL cholesterol did not differ significantly between the two groups. However, we observed significant differences in changes in total and LDL cholesterol at 6 months, with the AI+WL group having a slight increase and the PB+WL experiencing a reduction (Table 4).

**Table 4:** Change in metabolic profile at 3 and 6 months.

**Hormone**	**Change**	**PBO+WL**	**AI+WL**	***p***	**Adjusted p**
Hemoglobin A1c (%)	Δ 3 months	−0.2 ± 1.1	−0.4 ± 1.1	0.74	0.14
	Δ 6 months	0.3 ± 1.3	−0.3 ± 1.3	0.33	0.10
LDL (mg/dL)	Δ 3 months	4.4 ± 36.7	4.8 ± 18.8	0.98	0.95
	Δ 6 months	−30.9 ± 46.7	5.7 ± 22.7	0.06	**0.03**
HDL (mg/dL)	Δ 3 months	1.0 ± 4.6	1.7 ± 3.5	0.72	0.70
	Δ 6 months	1.2 ± 2.8	0.5 ± 6.1	0.81	0.85
Triglycerides (mg/dL)	Δ 3 months	−18.6 ± 70.3	18.3 ± 61.2	0.25	0.27
	Δ 6 months	2.4 ± 47.5	−0.2 ± 71.4	0.94	0.80
Total cholesterol (mg/dL)	Δ 3 months	2.5 ± 36.8	5.9 ± 21.9	0.81	0.82
	Δ 6 months	−33.2 ± 46.7	4.5 ± 30.9	0.09	**0.03**

*Results are presented as mean ± standard deviation; p values adjusted for baseline values. Bold p values indicate a significant between group difference*.

### Bone Mineral Density, Bone Microarchitecture, and Markers of Bone Turnover

Changes in total hip and femoral neck areal BMD measure by DXA did not differ between our two groups (Table 5). However, the AI+WL group experienced a mild but non-significant reduction in the lumbar spine BMD compared to the PBO+WL (*p* = 0.08). Analysis of bone microarchitecture showed a significant difference in the changes in tibial trabecular area after 6 months of intervention, with the PBO+WL experiencing a reduction compared to no change in the AI+WL (Table 6). This difference was lost when adjusted for baseline value. Similarly, we did not detect any difference in changes in failure load and stiffness of tibia and radius, with and without adjustment for age and WL. Overall, changes in the markers of bone turnover OC and CTX did not significantly differ between the AI+WL and PBO+WL groups at 3 months (OC: 17.6 ± 47.6 vs. −4.8 ± 22.2 ng/ml, *p* = 0.27; CTX: −0.6 ± 46.2 vs. −1.2 ± 15.1 ng/ml, *p* = 0.98) and 6 months (OC: 19.8 ± 62.1 vs. −1.9 ± 25.4 ng/ml, *p* = 0.40; CTX: 8.8 ± 36.8 vs. −3.8 ± 26.5 ng/ml, *p* = 0.77) of intervention.

**Table 5:** Change and % change in total hip and lumbar spine areal bone mineral density (BMD) at 6 months.

**Skeletal site**	**Change**	**PBO+WL**	**AI+WL**	***p***	**Adjusted *p***
Total hip BMD	Δ	−0.014 ± 0.040	−0.005 ± 0.032	0.63	0.66
	%	−1.452 ± 3.980	−0.456 ± 2.661	0.56	
Femoral neck BMD	Δ	−0.016 ± 0.027	0.007 ± 0.042	0.28	0.27
	%	−1.633 ± 2.915	0.823 ± 3.833	0.20	
Lumbar spine BMD	Δ	0.034 ± 0.052	−0.010 ± 0.041	0.09	0.15
	%	3.222 ± 4.893	−0.708 ± 3.321	0.08	
Trabecular bone score	Δ	0.025 ± 0.090	0.091 ± 0.104	0.22	0.19
	%	2.070 ± 7.724	8.071 ± 8.523	0.18	

*Results are presented as mean ± standard deviation; p value were adjusted for baseline values*.

**Table 6:** Change and % change in radial and Tibial bone microarchitecture by HR-pQCT at 6 months.

	**Change**	**Radius**				**Tibia**			
		**PBO+WL**	**AI+WL**	***p***	**Adjusted *p***	**PBO+WL**	**AI+WL**	***p***	**Adjusted p**
Total volumetric BMD	Δ	1.67 ± 4.24	−3.39 ± 11.18	0.32	0.44	4.12 ± 5.93	1.59 ± 3.68	0.38	0.38
	%	0.39 ± 1.34	−0.78 ± 2.81	0.37		1.20 ± 1.84	0.67 ± 1.36	0.58	
Cortical area	Δ	−0.22 ± 3.36	−2.56 ± 11.40	0.64	0.73	8.28 ± 12.09	−1.14 ± 4.54	0.07	0.11
	%	−0.25 ± 4.37	−1.79 ± 11.98	0.77		5.45 ± 8.54	−0.64 ± 2.52	0.08	
Cortical porosity	Δ	−0.00 ± 0.01	0.00 ± 0.01	0.72	0.22	−0.00 ± 0.01	−0.00 ± 0.01	0.88	0.99
	%	−0.98 ± 0.71	−1.05 ± 1.29	0.91		0.61 ± 23.09	−1.97 ± 15.92	0.82	
Cortical thickness	Δ	−0.04 ± 0.16	0.06 ± 0.09	0.16	0.18	0.07 ± 0.14	−0.01 ±.04	0.16	0.25
	%	−2.87 ± 13.84	5.70 ± 8.95	0.18		5.40 ± 10.80	−0.48 ± 2.11	0.15	
Trabecular area	Δ	−0.02 ± 2.00	5.69 ± 13.48	0.33	0.35	−17.40 ± 20.03	1.27 ± 4.80	**0.04**	0.06
	%	−0.02 ± 0.57	1.77 ± 4.07	0.31		−2.09 ± 2.20	0.12 ± 0.68	**0.03**	
Trabecular volumetric BMD	Δ	1.45 ± 2.79	−0.45 ± 3.97	0.34	0.39	−7.90 ± 14.64	0.96 ± 4.09	0.15	0.31
	%	0.64 ± 1.42	−0.08 ± 1.99	0.47		−6.49 ± 11.41	0.71 ± 2.48	0.13	
Trabecular number	Δ	0.04 ± 0.06	−0.01 ± 0.10	0.27	0.64	−0.06 ± 0.16	0.02 ± 0.12	0.33	0.38
	%	2.72 ± 3.73	−0.50 ± 6.14	0.28		−5.34 ± 13.15	1.91 ± 8.08	0.26	
Trabecular thickness	Δ	0.00 ± 0.01	−0.00 ± 0.01	0.06	0.07	0.01 ± 0.02	−0.00 ± 0.01	0.32	0.44
	%	0.74 ± 0.92	−0.64 ± 1.36	0.06		2.78 ± 7.66	−0.51 ± 1.75	0.29	
Trabecular separation	Δ	−0.01 ± 0.02	0.01 ± 0.03	0.28	0.57	0.08 ± 0.16	−0.01 ± 0.04	0.18	0.36
	%	−1.61 ± 2.43	0.95 ± 4.94	0.27		9.77 ± 20.59	−1.18 ± 5.99	0.21	
Stiffness	Δ	−2284 ± 3144	−4105 ± 7276	0.58	0.68	583 ± 7144	−3945 ± 12397	0.52	0.62
	%	−2.09 ± 3.29	−4.02 ± 6.42	0.52		0.36 ± 3.28	−1.31 ± 5.17	0.57	
Failure load	Δ	−130 ± 175	−271 ± 428	0.46	0.64	107 ± 372	−135 ± 608	0.49	0.58
	%	−2.16 ± 3.09	−4.63 ± 6.83	0.43		0.80 ± 2.9	−0.81 ± 4.62	0.54	

*Results are presented as mean ± standard deviation; p value adjusted for baseline value. Bold p values indicate a significant between group difference*.

### Adverse Events

Adverse events included: musculoskeletal pain (one for each of the following: neck and leg pain from a motor vehicular accident, shoulder pain, heel pain from a heel spur and back pain, non-cardiac-related chest pain); one subject had hot flushes which on further questioning has been there for years with no change in intensity; one subject had shortness of breath from unclear etiology; one had rashes which did not stop the subject from participation; one had diarrhea; one had deterioration in control of diabetes mellitus; one had constipation; one had suicidal ideation. None of the adverse events was related to the study drug. Adverse events are also listed in Supplement Table 2.1.

## Discussion

Our study demonstrates that although the combination of AI and WL is effective in reversing the hormonal profile of HHG compared to WL alone, it does not lead to greater improvements in muscle strength and symptoms of hypogonadism, contrary to our original hypothesis. On the other hand, we did not observe significant side effects from the therapy. This pilot project is the first comprehensive study on the effect of AI+WL compared to WL alone on clinical outcomes of hypogonadism, bone mechanical properties, and microarchitecture, in addition to changes in body composition and metabolic profile in the population of severely obese men with hypogonadism.

Obese patients above 40 years old with BMI≥30 kg/m^2^ have a higher risk of developing secondary hypogonadism (relative risk of 8.71) (30), postulated as due to the suppression of the hypothalamic-pituitary-gonadal axis made by hyperestrogenemia (5, 7). Weight loss improves the hormonal profile of obese hypogonadal men (8, 11), although these improvements are often lost due to weight regain (9). According to our preliminary results, severely obese men (BMI≥35 kg/m^2^) do not experience significant benefit from testosterone replacement: they in fact do not improve hormonal profile, symptoms, and body composition in the same way as individuals with a lower BMI (presented as an abstract at the annual Endocrine Society meeting in 2016). The use of AI has been tested as an alternative to treat obese men with hypogonadism (13, 14). Although these studies showed a significant improvement in hormonal profile, there was no amelioration in symptoms (12, 16, 17) and these trials have a quite short duration to detect changes in symptoms (12, 13). Moreover, in a study among elderly hypogonadal men, a possible adverse effect consisting of reduction in spine areal BMD after 1 year of AI was suggested, although spine trabecular volume was preserved (18). The only study exploring the effect of AI on clinical outcomes in severely obese men, did not find any significant difference in symptoms and side effects between subjects randomized to AI (letrozole) and those randomized to placebo (14). However, this study did not specifically include weight loss as part of the protocol, and since escalating doses of AI were used, participants ended-up on different doses at the conclusion of the trial (14). Our study showed a significant reversal of the hormonal profile typical of obesity induced HHG in the AI+WL group compared to WL alone. Changes in both T and E2 were significantly different between study arms with TT reaching a concentration in the midrange of normal in the AI+WL compared to the persistently subnormal levels in the PBO+WL group. Estradiol levels were also significantly lower in the AI+WL group compared to PBO+WL group at 6 months. However, contrary to our hypothesis, we were unable to detect greater improvements in muscle strength and symptoms of hypogonadism in the AI+WL compared to PBO+WL group. Considering that improvements in symptoms of hypogonadism and obesity-related quality of life can be achieved with WL alone (31), our finding was not completely surprising. Most of the explored symptoms are in fact related to increased body weight, and part of them can also be caused by hypogonadism and erectile dysfunction which improve as testosterone increases with weight loss (11).

Subjects included in the AI+WL group experienced a non-significant trend for higher weight loss, but a significantly higher reduction in total body fat mass compared to PBO+WL. Importantly, no significant differences in lean mass changes were observed. Weight loss-induced loss of muscle mass is always a concern when prescribing diet therapy, especially in the context of aging and hypogonadism, both of which are considered risk factors for sarcopenia (32, 33). Testosterone regulates myogenesis and lean mass (34). A study by Finkelstien et al. on men given GnRH to abolish endogenous testosterone production then treated with testosterone plus/minus AI demonstrated that testosterone is the primary regulator of lean mass while estradiol regulates fat mass in men (35). In addition, studies on hypogonadal men have shown a correlation between changes in testosterone levels and changes in lean mass (36). In our report despite a mean of 5 kg difference in weight loss (with AI+WL having greater weight loss compared to placebo), there was no difference in lean mass loss between the 2 groups. Estrogen is the main sex hormone regulating fat mass not only in women, but also in men (35). Men with estrogen lack from aromatase deficiency are obese and dyslipidemic (37). Estradiol in fact, besides promoting visceral lipolysis and subcutaneous adipogenesis (38), favors liver secretion of cholesterol into the bile (39). Hence, estrogen treatment results in improved body composition and metabolic profile in post-menopausal women (37). We recently reported that hypogonadal men and post-menopausal women at the opposite end of E2 spectrum have higher fat mass (40, 41). Furthermore, estrogen receptor alpha expression (ERα) decreases with increasing fat mass in men with low testosterone (41). We thus believe that men with high fat mass and hyperestrogenemia are resistant to estrogen action, as described by Schenider et al. (42) and supported by the lower expression of ERα in adipose tissues (41). It is possible that the reversal of hyperestrogenemia in severely obese hypogonadal men, improves estrogen sensitivity and favors higher fat mass loss, as experienced by our AI+WL group. Estrogen lack is considered a risk factor for lipid abnormalities and atherosclerosis in post-menopausal women (39) and aromatase inhibitor therapy in women with breast cancer may increase LDL and reduce HDL cholesterol, rising the cardiovascular risk in this population (43). In our study the significant difference in LDL changes between the two groups is mainly due to the substantial drop (27.5%) in LDL in the PBO+WL group which we believed as mostly due to weight loss. Although the AI+WL group experienced a rise in LDL, the magnitude of increase is minimal (an increase of 5.7 mg/dl or 5.1% from baseline) and will unlikely have a meaningful clinical impact. It is possible that the addition of AI attenuated the improvement in LDL induced by weight loss, but not enough to significantly increase the cardiovascular risk in this population, consistently with the few studies that reported no significant changes in lipid profile in men randomized to AI (15, 16).

Although AI and WL are both considered risk factors for osteoporosis, our study did not reveal any difference in changes in areal BMD at the lumbar spine, hip or forearm, as well as no differences in changes in volumetric BMD at the tibia and radius between the two groups. The absence of significant alteration in bone metabolism is supported by the lack of differences in changes of bone turnover markers between the two groups. Interestingly, trabecular bone area at the tibia was better preserved in the AI+WL group compared to the PBO+WL group. This finding could be either related to improvements in TT levels, responsible for maintenance of bone cross sectional area (19) or to improvements in estrogen sensitivity (41). The trabecular bone is in fact more sensitive to E2 fluctuation as compared to the cortical component (27). According to our finding, AI+WL therapy does not cause major side effects on bone health.

Because AI can increase T levels, we also evaluated its effect on the prostate by monitoring changes in PSA and in lower urinary tract symptoms by IPSS as part of safety outcomes. A small short-term study of 42 subjects showed a small but significant increase in PSA in men randomized to letrozole compared to placebo, but there was no report of an increase in associated symptoms (14). However, consistent with results from the few existing trials investigating the effect of AI on prostate markers (12, 16), our study did not detect significant differences in changes in PSA or IPSS.

Our study has several limitations. Because of study design (pilot research) sample size is small and the relatively of short duration of the intervention does not allow us to conclude whether the reported changes would persist in a longer trial and/or in a larger population of obese men with hypogonadism. In conclusion, our study demonstrates that AI+WL is an effective strategy able to reverse the hormonal profile of patients with obesity induced HHG although this was not associated with significant improvement in muscle strength and symptoms of hypogonadism. In addition, we showed that the addition of AI to WL is associated with greater reduction in total body fat without causing major side effects, such as lipid profile abnormalities and loss of lean and bone mass. Clinical trials with larger sample size and longer duration are needed to confirm our findings.

## Data Availability Statement

The datasets generated for this study are available on request to the corresponding author.

## Ethics Statement

The studies involving human participants were reviewed and approved by Baylor College of Medicine Review Board. The patients/participants provided their written informed consent to participate in this study.

## Author Contributions

GC and RA-V: conceptualization. GC, CT, CQ, and RA-V: formal analysis. GC, RC, BJ, SM, DV, and RA-V: investigation. GC, RC, CT, FV, CQ, BJ, SM, DV, and RA-V: writing, reviewing, and editing.

## Conflict of Interest

The authors declare that the research was conducted in the absence of any commercial or financial relationships that could be construed as a potential conflict of interest.
