# Supplementary material for: Aromatase Inhibitors Plus Weight Loss Improves the Hormonal Profile of Obese Hypogonadal Men Without Causing Major Side Effects
Source: Front Endocrinol (Lausanne). 2020 May 15;11:277. doi: 10.3389/fendo.2020.00277 (PMC7243137; doi:10.3389/fendo.2020.00277)
Supplement: Supplementary file 1 [file Data_Sheet_1.PDF]

✓Compare

Institutional Review Board for Baylor College of Medicine and Affiliated Hospitals

Protocol Number: H-36912
Status: Approved
Initial Submit Date: 5/1/2015
Approval Period: 1/2/2019 - 12/10/2019

Section Aa: Title & PI

A1. Main Title

AROMATASE INHIBITORS IN SEVERELY OBESE HYPOGONADAL MALE VETERANS

A2. Principal Investigator

Name:	REINA VILLAREAL	Phone:	713-794-7534
Id:	184419	Fax:	713-794-7771
Department:	MEDICINE: ENDOCRINOLOGY	Email:	rvillare@bcm.tmc.edu
Center:		Mail Stn:	BCM185

A3. Administrative Contact

Name:	DIANA NIETO	Phone:	713-7947920
Id:	Non-Baylor	Fax:	
		Email:	nieto.diana@va.gov
		Mail Stn:	

A3a. Financial Conflict of Interest

Does any member of study personnel (Investigator (including investigator's spouse and/or dependent children)) that are involved in the design, conduct, or reporting of the research have a Significant Financial Interest (SFI) that would reasonably appear to be affected by the research for which funding is sought and/or associated with an entity/business that would reasonably appear to be affected by the research?

No

Section Ab: General Information

A4. Co-Investigators

Name:	RUI CHEN	Phone:	713-798-0190
Id:	177323	Fax:	
Department:	MEDICINE: ENDOCRINOLOGY	Email:	ruic@bcm.tmc.edu
Center:		Mail Stn:	BCM620

Name:	BRYAN C. JIANG	Phone:	713-798-0190
Id:	177335	Fax:	
Department:	MEDICINE: ENDOCRINOLOGY	Email:	bjiang@bcm.tmc.edu
Center:		Mail Stn:	BCM620

Name:	DENNIS T. VILLAREAL	Phone:	713-794-7156
Id:	184416	Fax:	713-794-7771
Department:	MEDICINE: ENDOCRINOLOGY	Email:	villarea@bcm.tmc.edu
Center:		Mail Stn:	BCM185

Name:	GEORGIA COLLELUORI	Phone:	713-794-7156
Id:	187403	Fax:	

Department: MEDICINE: ENDOCRINOLOGY
Center:
Email: colleluo@bcm.tmc.edu
Mail Stn: BCM185

Name: FRANCESCA VIGEVANO
Id: 204819
Department: MEDICINE: ENDOCRINOLOGY
Center:
Phone: 713-578-4300
Fax: 713-798-8764
Email: vigevano@bcm.tmc.edu
Mail Stn: BCM185

Name: SANJAY NAVIN MEDIWALA
Id: 691884
Department: MEDICINE: ENDOCRINOLOGY
Center:
Phone: 713-791-1414
Fax: 713-794-7714
Email: mediwala@bcm.tmc.edu
Mail Stn: BCM185

Name: BIJU JOHNSON
Id: Non-Baylor
Institution: Michael DeBakey VAMC
Address: 2002 Holcombe Blvd
Phone: 7137948850
Fax:
Email: Biju.Johnson@va.gov

A5. Funding Source:

Baylor College of Medicine (Internal Funding Only)

A6a. Institution(s) where work will be performed:

BCM: Baylor College of Medicine
Michael E. DeBakey Veterans Affairs Medical Center

A6b. Research conducted outside of the United States:

Country:
Facility/Institution:
Contact/Investigator:
Phone Number: 8326592947

If documentation of assurances has not been sent to the Office of Research, please explain:

A7. Research Category:

A8. Therapeutic Intent

Does this trial have therapeutic intent?
Yes

Section B: Exempt Request

B. Exempt From IRB Review

Not Applicable

Section C: Background Information

BACKGROUND After age of 40, testosterone (T) production in men gradually decreases at a rate of 1.6% per year for total and to 2-3% per year for bioavailable T (5). Because of the age-related increase in sex hormone binding globulin, the magnitude of the decrease in bioavailable T in men is even greater than the decline in total T levels (6). This reduction in T production in men parallels the age-associated loss of muscle mass that leads to sarcopenia and impairment of function and the age-associated loss of bone mass that leads to osteopenia and fracture risk (7;8). Hypogonadism is a condition associated with multiple symptom complex including fatigue, depressed mood, osteoporosis, increased fat mass, loss of libido and reduced muscle strength, all of which deeply affect patient's quality of life (9). The prevalence of hypogonadismamong obese men ranges between 29.3% to 78.8% (2;10), with levels of androgens decreasing proportionately to the degree of obesity (11), This decline in T levels is exacerbated among obese patients due the suppression of the hypothalamic-pituitary-gonadal axis by hyperestrogenemia (12). The high expression of aromatase enzyme in the adipose tissue enhances the conversion of androgens into estrogens (E) which in turn exerts a negative feedback on hypothalamus and pituitary (1), inhibiting the production of gonadotropin-releasing hormone (GnRH), luteinizing hormone (LH) and follicle stimulating hormone (FSH) and, as a consequence, of T by the testis resulting in hypogonadotropic hypogonadism (HH). Considering the high aromatase expression in the adipose tissue, the administration of T among obese men with HH could increase the conversion of the substrate T to estradiol (E2) and fuels

the negative feedback on the hypothalamus and pituitary, producing a greater suppression of GnRH and gonadotropins. Accordingly, preliminary data from our ongoing study (CYP19A1 Gene and Pharmacogenetics of Response to Testosterone Therapy) showed no improvement in hypogonadal symptoms and lean mass in severely obese men (BMI higher than 35 kg/m2) after treatment with T, compared to subjects with lower BMI. In addition, we also have data from our lifestyle intervention in obese men showing statistically significant improvement in T levels after 1 year among patients randomized to the weight loss groups relative to the weight maintenance groups. However, the increase in T levels is barely above the cut-off of 300 ng/dl set by the Endocrine Society as criteria for diagnosis of hypogonadism and far from the mid-eugonadal range. Unfortunately, as this was not a study on hypogonadism, no data on changes in symptoms related hypogonadism were collected. Thus, men with obesity induced HH may benefit from other treatment strategies that target the pathophysiology of the disease. Weight loss intervention which improves hormonal and metabolic abnormalities related to obesity may also be considered a logical approach to improve obesity-induced HH. A metaanalysis showed reduction in E2 and increase in LH, free and total T in men after both lifestyle and bariatric surgery interventions (2). However, increased T levels induced by weight loss are often lost due to weight regain (3), which is very frequent among patients undergoing massive weight loss (13). One possible approach consists of the use of aromatase inhibitors (AI) to stop the conversion of T to E2 thereby interrupting the vicious cycle of E2 inhibition of the hypothalamic-pituitary-gonadal axis and restoring T production to normal levels. Increased T and reduced E2 levels have been reported in men with low levels of T after AI administration, even though very few studies investigated clinical outcomes (14).The only study that explored the effect of AI on clinical outcomes such as bone density, body composition, psychological function and exercise capacity in HH obese men was the one of Loves et al. (15). However, a definitive conclusion could not be darwn from this study due to study design, i.e. escalating doses of Letrozole starting at 2.5 mg/week were administrated until a serum total testosterone of 20 nmol/l was reached. At the end of the 6-month study, research participants were on varied doses of AIs. In other studies using anastrozole 1 mg daily in elderly patients, an increase in testosterone to the mid-eugonadal range was observed but there were no improvement in symptoms, likely because these patients were not necessarily obese. However, there were also minimal side effects on bone health among men(15). Among the studies along looking at the effect of AIs in men with hypogonadism, only one reported a mild decrease in bone density which was not accompanied by deterioration in bone quality (21). In addition, there were no significant effects on the changes in body composition, metabolic risk factors, hematocrit, urinary symptoms and prostate-specific antigen (PSA) (14, 21). Bone loss is a recognized side effect of AIs in women with breast cancer (17;18;19;20;27;28;29), however, negative side effect on bone metabolism does not appear to be a major problem. In addition, in contrast to hypogonadal patients who have low E2 production from reduced T production, HH obese men present high levels of circulating estrogens that could potentially prevent them from bone loss, estradiol being the main regulator of the male skeleton. Our preliminary data showed significantly higher levels of estradiol, bone density at the lumbar spine total hip, trochanter and intertrochanter in men with a BMI greater than 35 kg/m2 (ongoing study: CYP19A1 Gene and Pharmacogenetics of Response to Testosterone Therapy). In HH obese men with baseline values of E2 more than 40 pmol/l, no bone loss was demonstrated after 6 month of AI administration compared to placebo (15). Our data (under review in the European Journal of Endocrinology) showed that hypogonadal men with high aromatase activity were able to maintain higher BMD despite low circulating T, but had lower lean and higher truncal fat mass compared to those with lower aromatase activity. We believe that AI use could promote positive changes on hypogonadal symptoms and body composition in HH severely obese patients, acting at the physiopathology of the disease without necessarily causing bone loss as reported by Loves et al (15). Given the difficulties among massively obese patients in losing weight to a BMI of less than 35 together with the difficulty related to weight loss maintenance, the effect of AI in conjunction with weight loss should be considered as an attractive alternative to increase and maintain T levels to normal.

Section D: Purpose and Objectives

OBJECTIVES: 1. To evaluate the effect of AIs in combination with weight loss compared to weight loss alone on the changes in muscle strength, and symptoms of hypogonadism in massively obese men with hypogonadotropic hypogonadism 2. To evaluate the effect of AIs in combination with weight loss compared to weight loss alone on the changes in body composition and visceral adipose tissue in massively obese men with hypogonadotropic hypogonadism. 3. To evaluate the effect of AIs in combination with weight loss compared to weight loss alone on bone mineral density and bone quality in massively obese men with hypogonadotropic hypogonadism. We hypothesize that in severely obese men with HH, the combination of AI therapy and weight loss intervention would be an effective alternative treatment strategy in improving HH related signs and symptoms without the adverse effect on bone metabolism.

Section E: Protocol Risks/Subjects

E1. Risk Category

Category 2: Research involving greater than minimal risk, but presenting the prospect of direct benefit to the individual subjects.

E2. Subjects

Gender:
Male

Age:
Adult (18-64 yrs), Geriatric (65+ yrs)

Ethnicity:
All Ethnicities

Primary Language:
English

Groups to be recruited will include:
Patients

Which if any of the following vulnerable populations will be recruited as subjects?

Vulnerable populations require special protections. How will you obtain informed consent, protect subject confidentiality, and prevent undue coercion?

E3. Pregnant woman/fetus

Will pregnant women and/or fetuses (as described in 45 CFR 46 Subpart B) be enrolled in the research?
No

E4. Neonates

Will neonates of uncertain viability or nonviable neonates (as described in 45 CFR 46 Subpart B) be enrolled in the research?
No

E5. Children

Will children be enrolled in the research?
No

Section F: Design/Procedure

F1. Design

Select one category that most adequately describes your research:
c) Pilot

Discuss the research design including but not limited to such issues as: probability of group assignment, potential for subject to be randomized to placebo group, use of control subjects, etc.
See attachment entitled " F1 Design IRB 7.29"

Inclusion Criteria:
Inclusion criteria: we will enroll 35-65 years old severely obese (BM I= or above 35 kg/m2) male veterans with hypogonadotropic hypogonadism defined as low total testosterone (lower than 300 ng/dl) taken from the average of two morning blood samples obtained between 8:00 and 11:00 AM in different days, at least 2-3 days apart, with LH lower than 9 U/L, estradiol above 40 pmol/l, and normal FT4,TSH, prolactin, cortisol, ACTH and IGF1 levels. Subjects must be ambulatory, willing and able to provide written informed consent.

Exclusion Criteria:
Exclusion criteria:

1) clinical or biochemical evidence of pituitary or hypothalamic disease; 2) any ongoing illness that, in the opinion of the investigator, could prevent the subject from completing the study; 3) any medication known to affect gonadal hormone levels, steroid hormone-binding globulin levels or bone metabolism (e.g., androgens, estrogens, glucocorticoids, phenytoin, or bisphosphonates); any medication known to interfere with anastrozole metabolism (tamoxifen and estrogens). 4) diseases known to interfere with bone metabolism as osteoporosis, hyperparathyroidism, untreated hyperthyroidism, osteomalacia, chronic liver disease, renal failure, hypercortisolism, malabsorption and immobilization; patients with a Total T score lower than -2.0 at Lumbar Spine or Left Femur. 5) symptomatic prostate disease, prostate carcinoma or elevated serum PSA more than 4 ng/ml or >3 for subject with family history of prostate cancer among first degree relatives (needs urologic evaluation before admission into study); 6) hematocrit greater than 50%, untreated severe obstructive sleep apnea, severe lower urinary tract symptoms with International Prostate Symptom Score above 19; 7) documented heart failure, cardiovascular disease, liver disease; 8) excessive alcohol or substance abuse; 9) unstable weight (changes in weight more than ± 2 kg) during the last 3 months; 10) history of bariatric surgery 11) subjects with elevated liver enzymes as alanine transaminase (ALT), aspartate transaminase (AST), alkaline phosphatase (ALP) and bilirubin at more than twice the upper limit of normal.

F2. Procedure

Screening and baseline tests:

1. Medical, social and medication history, body weight and height data will be collected and a physical examination with a digital rectal examination (DRE) will be done (all are standard of care). 2. Blood testing will be obtained for fasting testosterone from two samples taken between 8-11 AM at 2 separate days and at least 2-3 days apart (standard of care). 3. Blood tests for comprehensive metabolic panel (CMP), fasting lipid panel, aspartate aminotransferase (AST), alanine aminotransferase (ALT), alkaline phosphatase (ALP), bilirubine, HbA1c, complete blood count (CBC), prostate specific antigen (PSA), luteinizing hormone (LH), follicle stimulating hormone (FSH), prolactin, thyroid stimulating hormone (TSH), insulin growth factor-1 (IGF1), free thyroxine (FT4), cortisol , adrenocorticotropic hormone (ACTH), 25-hydroxyvitamin D (25OHD), parathyroid hormone (PTH), estradiol, bone markers osteocalcin, and N-terminal telopeptide (NTx), and sex hormone binding globulin (SHBG). Blood obtained from an antecubital vein, will be processed immediately, and be stored in aliquots at -80C (standard of care). 4. Quantitative Androgen Deficiency in Aging Male (ADAM) questionnaire to assess for symptoms of hypogonadism and severity (25), International Index of Erectile Function (IIEF) questionnaire, Stanford physical activity 7 days recall questionnaire, SF-36 questionnaire to assess patient¿s quality of life, and the Impact of Weight on Quality of Life Questionnaire- Lite (IWQOL) to evaluate the effect of excess weight on quality of life will be all administered These questionnaires are mostly research although most of the questions in the questionnaires are part of the review of systems which is standard of care. 5. Muscle strength will be assessed through Biodex System 4 isokinetic dynamometer (Shirley, NY); peak torque for isokinetic and isometric knee extensor and flexor will be measured. During the testing, the participants will seat with their backs supported and hips positioned at 120° of flexion and secured to the seat of the dynamometer with thigh and pelvic straps. All tests will be performed on the right leg. Testing will be performed at an angular velocity of 60° per second. The best of the 3 maximal voluntary efforts for each of the knee flexion and extension will be used as the measure of absolute strength and report as peak torque at 60° in Newton-meter (N.m) units. This is research. 6. Areal Bone mineral density (BMD) will be assessed by dual energy x-ray absorptiometry (DXA), of the spine, hip, wrist and whole body, bone volumetric BMD and bone quality of the spine and hip will be measured by central quantitative computed tomography (QCT), bone volumetric BMD and bone quality of the peripheral skeleton will be measured by high-resolution peripheral quantitative computed tomography (HR-pQCT) on the left tibia, and body composition and visceral adipose tissue volume by DXA. Trabecular bone score (TBS) will be calculated from the spine BMD obtained by DXA. BMD by DXA is standard of care while the rest is research.

Follow-up testing (please see Table and Schedule of tests):

1. Medical, social and medication history, and physical examination, body weight and height will be repeated at 3 and 6 months. DRE will be repeated at 6 months. An additional physical examination (except for digital rectal exam) will be done after 2 weeks from the start of the intervention for safety monitoring. 2. The ADAM quantitative questionnaire, IIEF questionnaire, Stanford physical activity 7 days recall questionnaire, SF-36 questionnaire and the IWQOL will be administered at baseline, 3 and 6 months. 3. Muscle strength by Biodex will be assessed at baseline, 3 and 6 months (not standard of care). 4. Participants will be given instructions for keeping 7-day food intake records and provided with recording forms to estimate energy intake and nutrient composition. The food intake records will be analyzed using the Nutritionist IV computer program (N2 Computing, San Bruno, CA). The Stanford 7-Day Physical Activity Questionnaire will be used to quantify the time spent in different categories of activity during the previous 7 day period. Participants will also be asked to wear accelerometers for the whole intervention and to report daily data concerning the instrument usage. 5. Blood will be collected at 2 weeks a for testosterone, estradiol, LH and FSH, AST, ALT,ALP and bilirubin and at 3 and 6 months for repeat PSA, total testosterone, CBC, CMP, lipid panel, HbA1C, ALP, AST, ALT and bilirubin, markers of bone turnover, estradiol, SHBG, LH, FSH, 25-hydroxy vitamin D and PTH (all test are standard of care except for estradiol and SHBG). 6. Bone mineral density will be measured using DXA, QCT, and HR-pQCT at baseline and 6 months. TBS will be calculated at baseline and 6 months, whereas body composition and visceral adipose tissue volume will be measured at baseline, 3 and 6 months, both will be assessed using DXA. 7. At baseline, 3 and 6 month, blood will be drawn for extraction of DNA, which is part of the cell and os responsible for providing hereditary characteristics. The purpose of analyzing DNA is to determine whether hereditary characteristics are associated with obesity induced HHG and whether the response to weight loss and AI administration is affected by them

Section G: Sample Size/Data Analysis

G1. Sample Size

How many subjects (or specimens, or charts) will be used in this study?
Local: 23 Worldwide: 30

Please indicate why you chose the sample size proposed:
This is a pilot study with the primary objective of gathering preliminary feasibility and effectiveness data for the development of a bigger study. We plan to enroll 30 subjects for this study. In prior studies of patients with HH using sample sizes of 20-40, a significant increase in T levels was observed for those on AIs compared to those without AIs. In our proposed study, we anticipate a greater increase in T in our patient's randomized to AI over and above the increase resulting from weight loss alone by the same magnitude of increase in above studies which compared the effect of AI alone to those not on AI. Thus, a sample of 30 was chosen for this pilot study. Although the above studies did not include

changes in hypogonadal symptoms as outcomes, we aniticipate greater improvement in symptoms of hypogonadism among patients on AIs plus weight loss. It is highly likely that we may not be able to find a statisitcally significant difference between the AIs plus weight loss vs. weight loss alone group in our primary outcomes given this limited sample size, but our results would still provide important information for a bigger study if a trend is observed.

G2. Data Analysis

Provide a description of your plan for data analysis. State the types of comparisons you plan (e.g. comparison of means, comparison of proportions, regressions, analysis of variance). Which is the PRIMARY comparison/analysis? How will the analyses proposed relate to the primary purposes of your study?

Baseline characteristics between groups will be compared using independent t-test or Fisher¿s exact test. Percent changes in outcomes at 3 and 6-month time points will be compared between the 2 groups using repeated measures analysis of covariance adjusting for covariates such as age, ethnicity and medication use as appropriate. A p value of <0.05 will be considered significant. Simple correlations will be analyzed using Pearson correlation coefficient.

Given the limited sample size, it is possible that we many not find a significant difference in outcomes between the anastrozole+weight loss vs. weight loss alone but the information generated from this pilot study will still be useful if a trend is observed for future proposals or plan for additional recruitment to enhance the sample size.

Section H: Potential Risks/Discomforts

H1. Potential Risks/Discomforts

Describe and assess any potential risks/discomforts; (physical, psychological, social, legal, or other) and assess the likelihood and seriousness of such risks:

POTENTIAL RISKS AND DISCOMFORT We anticipate no psychological, social or legal risks beyond those of participation in health-related research in general. The potential risks of this study are small and primarily associated with the testing measures and anastrozole therapy as described in the next paragraphs: Risk related to aromatase inhibitors administration: available data regarding potential risks and discomfort due to the use of aromatase inhibitors refer to women (18;27-29). Furthermore, the lower levels of estradiol in postmenopausal women compared to men have to be taken into account. Bone loss was also reported primarily in women taking the medication for breast cancer(28;30). A report of bone loss was also reported in elderly men, not the population we are proposing in our study(19). A prior report showed no bone loss if patients are severely obese with estradiol levels over 40 pmol/L (10.9 pg/ml) which is the target population for this proposed project (15). Subject will be informed about the possible risks and discomfort and instructed to report all of them to the research team. The following are the side effect reported for women after AI treatment. a) Non severe side effect: weakness, headache; hot flashes; sweating; stomach pain; nausea; vomiting; loss of appetite; constipation; diarrhea; heartburn; joint; bone or muscle pain; breast pain; mood changes; depression; difficulty falling asleep or staying asleep; nervousness; dizziness; pain, burning, or tingling in the hands or feet; dry mouth; hair thinning. b) Severe side effects: chest pain; sore throat, cough, fever, chills, swollen glands, or other signs of infection; swelling, redness, or warmth in hand or arm; difficult, painful, or urgent urination; blurred vision or vision changes; yellowing of the skin or eyes; pain in the upper right part of the stomach; skin lesions, ulcers, or blisters; rash; hives; itching; shortness of breath; difficulty swallowing or breathing; swelling of the eyes, face, lips, tongue, throat, arms, hands, feet, ankles, or lower legs. Allergic reaction: Symptoms may include but not be limited to trouble breathing, fast heart rate, rash, dizziness, and swelling. Risks related to blood drawing: possible temporary pain and discomfort from the needle stick, occasional bruising and or bleeding at the site of needle insertion, sweating, feeling faint or lightheaded and in rare cases infection. There have been reported serious skin reactions (such as Stevens-Johnson Syndrome). Risk related to radiation (x-ray) risks: This research involves exposure to radiation from the dual energy x-ray absorptiometry (DXA) and high-resolution peripheral quantitative computed tomography (HR-pQCT) for bone mineral density and body composition measurements, as well as from spine X-rays. The amount of radiation from these tests when averaged over our entire body is less than 3% of the allowable annual dose to a radiation worker (for example, X-ray technicians). The risk from the radiation exposure in this study is too small to be measured and not more than the usual daily exposure. Risk related to lifestyle intervention: A modest muscle and bone density loss associated with weight loss can occur. However, this is prevented by exercise (26;31) (32) which we propose to combine with weight loss. A rare risk of weight loss is the formation of gallstone.

There are risks related to the loss of confidentiality. The health information that we may use, disclose or release for this research is past and present medical and mental health information, alcoholism or alcohol abuse information, medications, tests including diagnostic laboratory, pathology results, imaging such as x-rays, MRIs CT scans, dates of tests, demographic information such as name, date of birth, age, home address, phone number, last four SSN, race, dates of tests and test results, completed research questionnaires and records of study drug received. These information may be disclosed unintentionally in the course of the study.

Genetic research risk: There are risks of loss of privacy, getting insured, becoming employed, and stigmatization from results of genetic testing. There are some protections afforded by the Genetic Information Nondiscrimination Act (GINA). Participation in research may involve a loss of privacy or confidentiality, but personal information will be kept as confidential as possible. Representatives from the Baylor College of Medicine Human Research Protection Office, Michael DeBakey VA Medical Center and National Institute of Health will be permitted to access patient records.

There are risks of stress, emotional distress, and inconvenience associated with participating in aresearch study. Participants may experience emotional discomfortwhen answering some questions in the questionnaires. Participants will

be encouraged to discuss the importance and the need to answer these questions with a member of the research team. If they remain uncomfortable in answering these questions despite the above efforts, participants will be given the option not to answer them.

Unknown risks: The experimental treatments may have side effects that no one knows about yet including potential death. They may also have unknown risks to a fetus or embryo.

H2. Data and safety monitoring plan

Do the study activities impart greater than minimal risk to subjects?

Yes

NOTE: The answer to the questions in H2 requires the completion of the form: 'Section H – Data and Safety Monitoring Plan' as an attachment in Section S.

H3. Coordination of information among sites for multi-site research

Is the BCM Principal Investigator acting as the SPONSOR-INVESTIGATOR for this multi-site research?

No or Not Applicable

Is BCM the COORDINATING CENTER for this multi-site research?

No or Not Applicable

Section I: Potential Benefits

Describe potential benefit(s) to be gained by the individual subject as a result of participating in the planned work.

POTENTIAL BENEFITS: Patients may or may not benefit from participating in this study. Improvement in quality of life and hypogonadal symptoms including muscle strength, sexual function, mood, and body composition, may be experienced by subjects participating in this study. Weight loss and related metabolic improvement may also occur.

Describe potential benefit(s) to society of the planned work.

The information gained from this study may help identifying the best way of treatment for the obesity-related hypogonadotropic hypogonadism : its different physiopathology necessitates an effective therapy able to act the the root of the disease and thus improve hypogonadal symptoms and muscle strength. According to our preliminary data, this subgroup of hypogonadal obese subject do not benefit from testosterone therapy.

Do anticipated benefits outweigh potential risks? Discuss the risk-to-benefit ratio.

The benefits to the subjects studied in this research protocol, and to society at large, far surpass the risks. In addition to having weight loss, improvement of symptoms related to hypogonadism may result from a combination of weight loss and anastrozole in severely obese subjects who are likely not to respond to testosterone treatment. On the other hand among the side effects, erythrocytosis, increase in PSA and prostatic enlargement are well-recognized side effect of increase levels from testosterone administration. Furthermore, recent studies reported increase in the risks of myocardial infarction, stoke associated with testosterone treatment. Although we plan to monitor our subjects closely, these events have not been reported in studies using AIs. Bone loss has been reported in postmenopausal women with breast cancer and in elderly men hypothesized to be due to profound estrogen deficiency resulting from AI use. However, we do not foresee this as a problem for severely obese men who have elevated levels of estrogens at baseline. Thus, we believe that the benefits outweigh the risk with this treatment regimen which if found to be effective, may potentially become the standard of care for severely obese patients.

Section J: Consent Procedures

J1. Waiver of Consent

Will any portion of this research require a waiver of consent and authorization?

Yes

Please describe the portion of the research for which a waiver is required. (Example: chart review to determine subject eligibility)

Chart review to determine subject eligibility (for screening purposes only).

Explain why the research and the use or disclosure of protected health information involves no more than minimal risk (including privacy risks) to the individuals.

No 38 U.S.C. 7332 information (drug abuse, alcohol abuse, HIV infection and sickle cell anemia) will be used in this research study

Explain why the waiver will not adversely affect the privacy rights and the welfare of the research subjects.

We will review the patients charts in the electronic medical record system in the VA known as Computerized Patient

Record System (CPRS) in the same way as we review records of our clinical patients, the access to which is password protected. All members of the research team will be issued an individual password. We plan to protect identifiers from improper use or disclosure. Records that are queried will be stored in folders on VA computer space to which acess is restricted to personnel authorized by the VA Research and Development Committee, specifically the PI and study coordinator. Data will not be shared on hard drives of any workstation, or transmitted to any other facilty. All printing will be directed to a printer in a private office, not to common work areas. Once permission has been obtained from their providers to contact patients, files containing identifiers will be maintained for as long as it takes to mail out solicitation letters and fully recruit for the study.

Any information that is collected as part of the research including PHI will not be used or disclosed to a third party except as required by law or permitted by a HIPAA authorization. The identifiers will be destroyed at the earliest opportunity according to VA guidelines

Explain why the research could not practicably be conducted without the waiver and could not practicably be conducted without access to and use of the protected health information.

There are a large number of patients in the database and it is not possible to identify whether they might meet basic eligibility criteria without first accessing PHI and we would not be able to obtain HIPAA authorization from all of these patients prior to enrollment.

Describe how an adequate plan exists in order to protect identifiers from improper use and disclosure.

Access to the CPRS at the MEDVAMC is limited to individuals who are involved in patient care or human research and is password protected. Paper documents will be placed in double locked doors with only members of research team are allowed access. In addition, no names will be used in any communication or publication resulting from the study. Please also see our response to (a) above.

Describe how an adequate plan exists in order to destroy identifiers at the earliest opportunity consistent with conduct of the research, unless there is a health or research justification for retaining the identifiers or such retention is otherwise required by law.

Any files that contain identifiers for the specific purpose of screening and determining eligibility (as part of this waiver of consent) will be destroyed at the earliest opportunity (i.e. when no longer used to determine eligibility and recruit for the study) according to VA guidelines.

Describe how adequate written assurances exist in order to ensure that the PHI will not be reused or disclosed to (shared with) any other person or entity, except as required by law, for authorized oversight of the research study, or for other research for which the use or disclosure of the PHI would be permitted under the Privacy Rule.

Names will not be used in any communication or published reports about this study. In the event that samples had to be shared with other laboratories, coded samples will be transferred without identifiers.

Information from health records such as diagnoses, progress notes, medications, lab or radiology findings, etc.

Yes

Specific information concerning alcohol abuse:

No

Specific information concerning drug abuse:

No

Specific information concerning sickle cell anemia:

No

Specific information concerning HIV:

No

Specific information concerning psychiatry notes:

No

Demographic information (name, D.O.B., age, gender, race, etc.):

Yes

Full Social Security #:

No

Partial Social Security # (Last four digits):

Yes

Billing or financial records:

No

Photographs, videotapes, and/or audiotapes of you:

No

Other:
No

Will additional pertinent information be provided to subjects after participation?
No

If No, explain why providing subjects additional pertinent information after participation is not appropriate.
No 38 U.S.C. 7332 information (drug abuse, alcohol abuse, HIV infection and sickle cell anemia) will be used in this research study

J1a. Waiver of requirement for written documentation of Consent

Will this research require a waiver of the requirement for written documentation of informed consent?
No

J2. Consent Procedures

Who will recruit subjects for this study?
PI
PI's staff

Describe how research population will be identified, recruitment procedures, any waiting period between informing the prospective participant and obtaining consent, steps taken to minimize the possibility of coercion or undue influence and consent procedures in detail.

We will recruit primarily male veterans attending the Endocrine, Primary Care and Urology Clinics at the Michael DeBakey VA Medical Center, Houston, TX. Active advertisement through posters and flyers, and direct mailings of printed materials to potential subjects will be done. Potential subjects will be identified from direct referral by the attending physicians of patients who have testosterone levels of lower than 300 ng/ml and a BMI= or higher than 35 kg/m2 under the inclusion criteria. The records will be reviewed for exclusion criteria. A member of the staff will then send a letter asking each primary care physician (PCP) for permission to contact his or her eligible patients. The PCP will respond by checking the names of those appropriate for this study. Once the provider¿s permission is obtained, a letter from the PCP will be sent to the patient describing the study and introducing the investigators. Interested patients will be asked to return a postcard.

Individuals who express an interest in participation will undergo a brief (~10 min) telephone interview by a member of the research team. All individuals who express an interest in participating and meet the preliminary inclusion criteria will be invited to visit our facility and discuss their potential participation in greater detail. During a 60 min long orientation session with members of the research team, detailed information will be provided regarding the aims of the study, and all of the tests and measurements that they will undergo if they participate in the study. Verbal and written information about the potential benefits and risks of the study will be provided; their questions will be answered and any concerns that they have will be addressed. If the individual is interested in participating, a screening evaluation will be scheduled. Prior to enrollment and randomization, the volunteers will undergo a detailed medical history and physical examination, and a clinical laboratory testing after a proper consent has been obtained in writing. During this session they will have the nature and purpose of the study explained to them again, discuss their reasons and motivation for participation in the research to determine whether they are realistic and discuss any potential problems, that might interfere with participation and have their questions answered. Informed consent to participate in the study will be obtained in writing by one of the investigators before any tests or measurements are performed. Data which include HIPAA identifiers such as names, date of birth or age, dates of tests and medical record number (first letter of last name plus last 4 digits of social security number) will be collected.

Each subject will be informed that their participation in the study is completely voluntary and they may withdraw by telling the study team that they are no longer interested in participating in the study or they may send in a withdrawal letter. They will also be informed that their choice will not at any time affect the commitment of their health care providers to administer care and that there will be no penalty or loss of benefits to which they are otherwise entitled.

For the Waiver of Consent for Subject Identification, please see the attached document entitled "J2- Waiver for Identification".

Are foreign language consent forms required for this protocol?
No

J3. Privacy and Intrusiveness

Will the research involve observation or intrusion in situations where the subjects would normally have an expectation of privacy?
No

J4. Children

Will children be enrolled in the research?
No

J5. Neonates

Will non-viable neonates or neonates of uncertain viability be involved in research?
No

J6. Consent Capacity - Adults who lack capacity

Will Adult subjects who lack the capacity to give informed consent be enrolled in the research?
No

J7. Prisoners

Will Prisoners be enrolled in the research?
No

Section K: Research Related Health Information and Confidentiality

Will research data include identifiable subject information?
Yes

Information from health records such as diagnoses, progress notes, medications, lab or radiology findings, etc.
Yes

Specific information concerning alcohol abuse:
Yes

Specific information concerning drug abuse:
Yes

Specific information concerning sickle cell anemia:
No

Specific information concerning HIV:
No

Specific information concerning psychiatry notes:
No

Demographic information (name, D.O.B., age, gender, race, etc.):
Yes

Full Social Security #:
No

Partial Social Security # (Last four digits):
Yes

Billing or financial records:
No

Photographs, videotapes, and/or audiotapes of you:
No

Other:
No

At what institution will the physical research data be kept?
Hard copies of the research data will be maintained at the MEDVAMC building 110, and will be stored in a locked office (room 240) inside a locked file cabinet, located in a restricted area.

How will such physical research data be secured?
Only the PI and research staff will have access to the room and cabinet where the information will be stored and they will take care of locking both at all time of usage.

At what institution will the electronic research data be kept?

Electronic data will be stored on the secure MEDVAMC S drive (restricted shared drive permission) in a subfolder of the main research folder.

The exact location of the electronic data is S:\Research-Data\Villareal, Reina\H-36912

Database location will be on a VA server

Such electronic research data will be secured via BCM IT Services- provided secured network storage of electronic research data (Non-Portable devices only):

No

Such electronic research data will be secured via Other:

Yes, (describe below):
Electronic data will be stored on the secure MEDVAMC S drive (restricted shared drive permission) in a subfolder of the main research folder .

The exact location of the electronic data is S:\Research\Villareal, Reina\H-36912

Database location will be on a VA server

Will there be anyone besides the PI, the study staff, the IRB and the sponsor, who will have access to identifiable research data?

Yes, identify the classes of the persons:
People who ensure quality from the institutions where the research is being done, federal and other regulatory agencies will have access to all of the research data such as Food and Drug Administration (FDA) and Data Monitoring Committee (DMC). The purpose of collecting information covered under 38 U.S.C. 7332 is to conduct scientific research and no personnel involved in this study will identify, directly or indirectly, any individual patient or subject in any report of such research.

Please describe the methods of transmission of any research data (including PHI, sensitive, and non-sensitive data) to sponsors and/or collaborators.

Data will not leave the MEDVA.

Will you obtain a Certificate of Confidentiality for this study?

No

Please further discuss any potential confidentiality issues related to this study.

Research records, including identifiers will be destroyed 6 years after cutoff (at the end of the fiscal year) after completion of the research project, but may be retained longer if required by other federal regulations or sponsor archive requirement.

Section L: Cost/Payment

Delineate clinical procedures from research procedures. Will subject's insurance (or subject) be responsible for research related costs? If so state for which items subject's insurance (or subject) will be responsible (surgery, device, drugs, etc). If appropriate, discuss the availability of financial counseling.

Subjects are not responsible for any costs related to participation in the research.

If subjects will be paid (money, gift certificates, coupons, etc.) to participate in this research project, please note the total dollar amount (or dollar value amount) and distribution plan (one payment, pro-rated payment, paid upon completion, etc) of the payment.

Dollar Amount:

60

Distribution Plan:

Subjects will be paid 20$ at baseline, 3 and 6 months. Total payment for completing the study will be 60$ per patient.

Section M: Genetics

How would you classify your genetic study?

DNA diagnostic study

Discuss the potential for psychological, social, and/or physical harm subsequent to participation in this research. Please discuss, considering the following areas: risks to privacy, confidentiality, insurability, employability, immigration status, paternity status, educational opportunities, or social stigma.

There is an associated genetic risk if the genetic information is leaked which may affect a subjects insurability. However, all of the patients are veterans getting medical care at the Michael DeBakey VA Medical Center whose benefits will not terminate with any breach of genetic information. In addition, since the type of genetic variations that this study is investigating are common genetic variations in the population, we foresee no consequences on the patients employablity, immigration status, educational opportunities or social stigma and not useful in establishing paternity.

Will subjects be offered any type of genetic education or counseling, and if so, who will provide the education or counseling and under what conditions will it be provided? If there is the possibility that a family's pedigree will be presented or published, please describe how you will protect family member's confidentiality?

No, subjects will not be offered any type of genetic education or counseling. There is not the possibility that a family's pedigree will be presented or published. The results of the genetic testing will not be given to the subjects. As the patients blood sample will not be linked to the patients identity, we plan to match the subjects identity with their DNA results with a study code assigned to each subjects DNA results which can only be interpreted with a special decrypting file.

Section N: Sample Collection

SAMPLE: Other: blood

What is the purpose of the sample collection?

Blood samples will be collected for 1) medical screening to determine eligibility for the study (see inclusion/exclusion criteria, 2) to monitor blood levels of testosterone, estradiol, LH, FSH and side effects on blood count and PSA, 3) overall safety monitoring while in the study

For blood draws, specify the amount drawn, in teaspoons, at each visit and across the course of the subjects entire participation time.

A total of 4 tablespoons of blood at baseline and 6 months, and 3 tablespoons at 2 weeks and at 3 months. Total amount of blood collected for participating in the study 14 tablespoons.

Is there the possibility that cell lines will be developed with this sample?No

Sample will be obtained from:

Clinical Labs, Research Labs

Will the sample be stripped of identifiers?

No

If sample will be released outside the hospital:

Will sample be released to anyone not listed as an investigator on the protocol? Will the information be identifiable, coded or de-identified?

Yes, to BCM collaborators. All samples will be coded using random numbers not linked to SSN.

Will sample material be sold or transferred to any third parties? Will the information be de-identified?

No.

If sample will be banked for future use:

Where will the sample be banked and for how long?

At the Michael DeBakey VA Medical Center for 10 years

Does the banking institution have an approved policy for the distribution of samples?

Yes.

If the entire sample will NOT be used during the course of this research study:

Will the remaining tissue be discarded? If not what will be done with the remaining sample after study completion and how long will the sample be kept?

We will keep the samples for 10 years after study completion after which they will be discarded.

Will samples be made available to the research subject (or his/her medical doctor) for other testing?

No

If a subject withdraws from the study:

Will subject have the option to get the remaining portion of their sample back?

No

Will samples be destroyed? If not, will they be kept anonymously? What will happen to the sample if the subject revokes authorization?

If the patient withdraws from the study, samples will not be destroyed and will be stored anonymously. If the subject revokes authorization, the samples will be destroyed or discarded.

Will data obtained from their sample be deleted? What will happen to the sample if the subject revokes authorization?

For those who withdraw from the study, we will use the data generated from the samples. However, for subjects who revoked authorization, their data will be deleted and samples destroyed or discarded.

Will study data or test results be recorded in the subject's medical records?

Yes

Will results of specific tests and/or results of the overall study be revealed to the research subject and or his/her doctor?

Standard test results that are done at VA lab and have clinical use are reported in CPRS, or Computerized Patient Record System, and thus they are automatically included in the subjects medical records (i.e. in CPRS, which is accessible to the vets primary provider). Any abnormal findings will be shared with the subject¿s primary care physicians for further evaluation and appropriate treatment through the CPRS which are shared with their VA primary care physicians. Depending on the urgency of the abnormal findings, the PI may directly contact the primary care provider by phone to discuss the abnormal findings

Please identify all third parties, including the subject's physician, to receive the test results.

Participants treating physicians.

SAMPLE: Blood

What is the purpose of the sample collection?

1) DNA collection to investigate the impact of the genetic profile on the response to anastrazole and/or weight loss intervention.

For blood draws, specify the amount drawn, in teaspoons, at each visit and across the course of the subjects entire participation time.

A total of 1 tablespoons of blood at baseline, 3 months and 6 months. Total amount of blood collected for genetic testing for participating in the study is 3 tablespoons.

Is there the possibility that cell lines will be developed with this sample?No

Sample will be obtained from:

Research Labs

Will the sample be stripped of identifiers?

No

If sample will be released outside the hospital:

Will sample be released to anyone not listed as an investigator on the protocol? Will the information be identifiable, coded or de-identified?

Yes, to BCM collaborators. All samples will be coded using random numbers not linked to SSN.

Will sample material be sold or transferred to any third parties? Will the information be de-identified?

No

If sample will be banked for future use:

Where will the sample be banked and for how long?

At the Michael DeBakey VA Medical Center for 10 years

Does the banking institution have an approved policy for the distribution of samples?

Yes

If the entire sample will NOT be used during the course of this research study:

Will the remaining tissue be discarded? If not what will be done with the remaining sample after study completion and how long will the sample be kept?

We will keep the samples for 10 years after study completion after which they will be discarded.

Will samples be made available to the research subject (or his/her medical doctor) for other testing?

No

If a subject withdraws from the study:

Will subject have the option to get the remaining portion of their sample back?

No

Will samples be destroyed? If not, will they be kept anonymously? What will happen to the sample if the subject revokes authorization?

If the patient withdraws from the study, samples will not be destroyed and will be stored anonymously. If the subject revokes authorization, the samples will be destroyed or discarded.

Will data obtained from their sample be deleted? What will happen to the sample if the subject revokes authorization?

For those who withdraw from the study, we will use the data generated from the samples. However, for subjects who revoked authorization, their data will be deleted and samples destroyed or discarded.

Will study data or test results be recorded in the subject's medical records?

Yes

Will results of specific tests and/or results of the overall study be revealed to the research subject and or his/her doctor?

Standard test results that are done at VA lab and have clinical use are reported in CPRS, or Computerized Patient Record System, and thus they are automatically included in the subjects medical records (i.e. in CPRS, which is accessible to the vets primary provider). Any abnormal findings will be shared with the subject¿s primary care physicians for further evaluation and appropriate treatment through the CPRS which are shared with their VA primary care physicians. Depending on the urgency of the abnormal findings, the PI may directly contact the primary care provider by phone to discuss the abnormal findings.

Please identify all third parties, including the subject's physician, to receive the test results.

Participants treating physicians.

Section O: Drug Studies

Does the research involve the use of ANY drug* or biologic? (*A drug is defined as any substance that is used to elicit a pharmacologic or physiologic response whether it is for treatment or diagnostic purposes)

Yes

Does the research involve the use of ANY gene transfer agent for human gene transfer research?

No

O1. Current Drugs

Drug : Arimidex 1 mg (anastrazole 1 mg tablet for oral use)

Is this study placebo-controlled?

Yes

If yes, be sure that you justify the use of the placebo for this research in the space below.

The placebo controlled trial is required to evaluate which is the best approach for the treatment of hypogonadotropic hypogonadism in severely obese men: life style intervention induced weight loss alone (placebo group) or life style intervention induced weight loss combined with anastrazole therapy (active group). IND # 126696

Will the research involve a radioactive drug that is not approved by the FDA?

No

Section P: Device Studies

Does this research study involve the use of ANY device?

No

Section Q. Consent Form(s)

None

Section R: Advertisements

Mode of Advertising: Bulletin Board

Exact language of Advertisement:

Below is the exact language of the advertisement for the study: This study is looking at the effect of weight loss alone or in combination with anastrozole, a medication which may increase testosterone levels, on muscle strength and symptoms related to low circulating testosterone. VOLUNTEERS MUST: - Be a male veteran - Be age 35-65 - Be obese - Not have prostate cancer - Not have a history of bariatric surgery - Not be on testosterone DURATION and LOCATION- Participation in the study will be 6 months. The study is conducted by Baylor College of Medicine and the Michael E. DeBakey VA Medical Center. The study will take place at the Michael E. DeBakey, Houston VA. INTERVENTION- Participants will take either the active drug (anastrozole) or placebo in addition to participation in a weight loss program (diet+exercise) TEST/PROCEDURES - Include phone screening, orientation session, medical examination, strength testing, bone mineral density and body composition testing and simple blood testing. COMPENSATION- Volunteers may receive up to $60.00. PI: Dr. Reina Villareal, M.D. 713-7947534 or 713-7911414 extension #24084

Mode of Advertising: Other: Flyers

Exact language of Advertisement:

Below is the exact language of the advertisement for the study: Testosterone study

This study is looking at the effect of weight loss alone or in combination with anastrozole, a medication which may increase testosterone levels, on muscle strength and symptoms related to low circulating testosterone.

VOLUNTEERS MUST: - Be a male veteran - Be age 35-65 - Be obese (BMI >35) - Not have prostate cancer - Not have a history of bariatric surgery - Not be on testosterone

DURATION - Participation in the study will be 6 months. The study is conducted by Baylor College of Medicine and the Michael E. DeBakey VA Medical Center. The study will take place at the Michael E. DeBakey, Houston VA.

INTERVENTION- Participants will take either the active drug (anastrozole) or placebo in addition to participation in a weight loss program (diet+exercise)

TEST/PROCEDURES - Include phone screening, orientation session, medical examination, strength testing, bone mineral density and body composition testing and simple blood testing.

COMPENSATION- Volunteers may receive up to $60.00.

PI: Dr. Reina Villareal, M.D. 713-7947534 or 713-7911414 extension #24084

Mode of Advertising: BCM Clinical Trials Website

Exact language of Advertisement:

Below is the exact language of the advertisement for the study: This study is looking at the effect of weight loss alone or in combination with anastrozole, a medication which may increase testosterone levels, on muscle strength and symptoms related to low circulating testosterone. VOLUNTEERS MUST: - Be a male veteran - Be age 35-65 - Be obese - Not have prostate cancer - Not have a history of bariatric surgery - Not be on testosterone DURATION and LOCATION- Participation in the study will be 6 months. The study is conducted by Baylor College of Medicine and the Michael E. DeBakey VA Medical Center. The study will take place at the Michael E. DeBakey, Houston VA. INTERVENTION- Participants will take either the active drug (anastrozole) or placebo in addition to participation in a weight loss program (diet+exercise) TEST/PROCEDURES - Include phone screening, orientation session, medical examination, strength testing, bone mineral density and body composition testing and simple blood testing. COMPENSATION- Volunteers may receive up to $60.00. PI: Dr. Reina Villareal, M.D. 713-7947534 or 713-7911414 extension #24084
