# Supplementary material for: Aromatase Inhibitors Plus Weight Loss Improves the Hormonal Profile of Obese Hypogonadal Men Without Causing Major Side Effects
Source: Front Endocrinol (Lausanne). 2020 May 15;11:277. doi: 10.3389/fendo.2020.00277 (PMC7243137; doi:10.3389/fendo.2020.00277)
Supplement: Supplementary file 2 [file Data_Sheet_2.docx]

**Supplement 2: Questionnaires**

1. **qADAM**

1. **IIEF-5**

1. **IWQOL**

1. **IPSS**

1. **PAR**

| **Table Supplement 2.1. List of Adverse events and Serious Adverse Events** | | |
| --- | --- | --- |
| **Date of event** | **Body System** | **Participant ID** |
| 11/7/2016 | Hot Flashes (AE) | H-36912-033 |
| 12/7/2016 | Shortness of Breath (AE) | H-36912-037 |
| 12/15/2016 | Left Shoulder Pain (AE) | H-36912-039 |
| 1/4/2017 | Skin Rush (AE) | H-36912-046 |
| 1/27/2017 | Diarrhea (AE) | H-36912-051 |
| 2/7/2017 | Heel Pain (AE) | H-36912-038 |
| 2/7/2017 | Uncontrolled DM (AE) | H-36912-042 |
| 3/3/2017 | Constipation (AE) | H-36912-051 |
| 3/9/2017 | Low Back Pain (SAE) | H-36912-046 |
| 3/20/2017 | Suicidal Ideation (SAE) | H-36912-034 |
